# The effectiveness of financial intervention strategies for reducing caesarean section rates: a systematic review

**DOI:** 10.1186/s12889-019-7265-4

**Published:** 2019-08-09

**Authors:** Yushan Yu, Feili Lin, Weizhen Dong, Haohan Li, Xiangyang Zhang, Chun Chen

**Affiliations:** 1 School of Public Health and Management, Wenzhou Medical University, Tongren, Building 7B304, Wenzhou Medical University Chashan campus, Wenzhou, 325035 China; 2 Department of Scientific Research and Education, Cancer Hospital, Chinese Academy of Science, Hefei, 230031 Hefei China; 3 Department of Preventive Medicine, Medical School of Shihezi University, Shihezi, 832003 China; 4 Department of Sociology and Legal Studies, University of Waterloo, 200 University Avenue West, Waterloo, Ontario N2L 3G1 Canada; 5 Zhongshan Hospital Fudan University, Shanghai, 200032 China; 6 First Affiliated Hospital of Wenzhou Medical University, Wenzhou, 325000 China

**Keywords:** Financial intervention, Caesarean section rate, CDMR

## Abstract

**Background:**

The increasing trend of Caesarean section (CS) in childbirth has become a global public health challenge. Previous studies have proposed financial intervention strategies for reducing CS rates by limiting caesarean delivery on maternal request (CDMR). This study synthesizes such strategies while evaluating their effectiveness.

**Methods:**

The sources of data for this study are Cochrane Library, PubMed, EMBASE, and CINAHL. The publication period included in this study is from January 1991 to November 2018. The financial intervention strategies are divide into two categories: healthcare provider interventions and patient interventions. Risk of Bias in Non-randomized Studies - of Interventions (ROBINS-I) was employed to assess the risk of bias of included studies. The outcome of each study was evaluated with Grades of Recommendation, Assessment, Development and Evaluation (GRADE) through the GRADEpro Guideline Development Tool software.

**Results:**

Nine studies were included in this systematic review: five with high certainty evidence (HCE), three with moderate certainty evidence (MCE), and one with low certainty evidence (LCE). Of the nine studies, seven are centered on the effect of provider-side interventions. Three of the HCE studies found that the diagnosis-related group payment system, risk-adjusted capitation, and equalizing fee for both facilities and physicians were effective intervention strategies. One HCE and one MCE study showed that only equalizing facility fees between vaginal and CS deliveries in healthcare service settings had no significant effect on reducing the CS rate. The MCE study showed that case payment had a negative effect on reducing the CS rates. One LCE study revealed that the effect of a global budget system was uncertain, and one HCE and one MCE study focused on combining both provider and patient-side interventions. However, equalizing fees for vaginal and CS deliveries and a co-payment policy for CDMRs failed to reduce the CS rate.

**Conclusions:**

The effectiveness of risk-adjusted payment methods appears promising and should be the subject of further research. Financial interventions should consider stakeholders’ characteristics, especially the personal interests of doctors. Finally, high-quality randomized control trials and comparative studies on different financial intervention methods are needed to confirm or refute previous studies’ outcomes.

**Electronic supplementary material:**

The online version of this article (10.1186/s12889-019-7265-4) contains supplementary material, which is available to authorized users.

## Background

The increasing trend of Caesarean section (CS) in childbirth has become a global public health challenge. Although the World Health Organization (WHO) is no longer recommending any specific CS rate, it has continuously warned that the rapid increase of CS rate should not be ignored, and has emphasized the need to avoid unnecessary CS worldwide [1]. In fact, that CS rate has increased from 19.5% (2000) to 27.2% (2014) in developed countries and from 13.1% (2000) to 20.9% (2014) in middle-income countries [2, 3]. Some less developed countries, such as Uganda and Kenya, are also experiencing a CS rate increase trend, although their growth rate is much slower than that of wealthier countries [4]. Unnecessary CS, or caesarean delivery on maternal request (CDMR) is the main cause of high CS rates [5]. The major concern with the increasing rates due to CDMR is that CS is associated with many short-term and long-term risks [6, 7] such as increased risk of asthma and obesity in children and increased risk of placenta previa and uterine rupture for mothers. Furthermore, high CS rates place a heavy burden on healthcare resources, which affects healthcare access equity [8, 9].

Therefore, different countries’ governments and their respective healthcare sectors have developed and adopted various intervention strategies for containing and reducing CS rates, particularly by limiting the use of CDMR. Such intervention strategies include professional, financial, and regulatory ones. Healthcare authorities and managed care organizations have primarily explored the aspects of financial interventions to contain and reduce the CS rates by controlling unnecessary CS [10–14]. Financial interventions are external motivations that intend to change the behavior of the demand or the supply side through monetary incentives [15]. Some studies showed that financial interventions had a positive effect on promoting a variety of healthcare services, such as improving outcomes in outpatient behavioral treatments [16], enhancing warfarin adherence [17], maintaining smoking cessation [18], and increasing the utilization of vaccinations [15].

A CS is a service provided in medical care settings. In theory, financial intervention strategies could influence the behaviors of doctors and mothers and have a direct or indirect effect on the rate of CS deliveries. Recently, researchers found evidence that non-clinical interventions reduced the rate of unnecessary CS.

We identified seven related reviews published in the last 9 years [19–25], which addressed a range of non-clinical strategies intended to reduce CS births, including educational interventions [20, 21, 23, 25], organizational interventions [20, 21, 23, 24], regulatory interventions [21, 23], audits and feedback [19–21, 23], practice guidelines [20, 21, 23], and financial interventions [20, 21, 23]. Some financial interventions, such as fee equalizing and financial reimbursement strategies, were discussed; however, the findings were conflicting. Thus, we argue that it is urgent and critical to identify the effectiveness of various financial interventions in reducing CS rates, because financial incentives are a major driver in modern society irrespective of a country’s income level.

Thus, the objectives of this systematic review are: (1) to determine the main financial intervention strategies developed, (2) to evaluate the effectiveness of these strategies, and (3) to synthesize relevant knowledge for policymakers to formulate financial interventions for reducing CS rates. Our review examines search strategies, study eligibility criteria, and the criteria for assessing the certainty of evidence.

## Method

### Search strategy

**Fig. 1:**
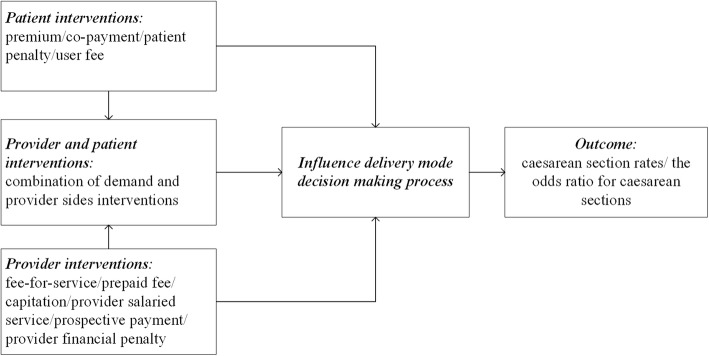
Financial interventions and the utilization of caesarean sections

We conducted a systematic search of English language CS rate relevant articles in the following electronic databases: Cochrane Library (1991 to November 2018), MEDLINE/PubMed (1948 to November 2018), EMBASE (1947 to November 2018), and CINAHL (1982 to November 2018) Additional file 1. We first searched these electronic databases using different combinations of search terms as shown in Fig. 1. Then, we conducted an additional search by screening the reference lists from the selected literature.

### Inclusion criteria

The inclusion criteria for this review consist of the following:

- Time range: Papers published between January 1991 to November 2018. This restriction was to ensure that they accurately represent financial interventions developed in recent decades.
- Types of studies: The included studies are randomized controlled trials, controlled clinical trials, cohort analytics (two groups: pre and post), cohort (one group pre + post: before and after), and interrupted time series (ITS) in which the intervention time was clearly defined, and there were at least three observations over time.
- Participants: The study participants are pregnant women and healthcare providers who work with expectant mothers. Studies on patients with particular conditions or specific risk factors (e.g., human immunodeficiency virus, pregnancy complications, preeclampsia, diabetes, obesity, hepatitis B, and herpes simplex virus) are excluded.
- Types of financial interventions: Financial interventions can be classified into two main types: provider and patient interventions (Fig. 1). The former includes fee-for-service, prepaid fee, capitation, provider salaried service, prospective payment, and provider financial penalty, while the latter includes premium, co-payment, patient penalty, and user fees [26]. This study’s focus is on the financial interventions that aim to reduce the CS rate.
- Types of outcomes: CS rates and the CS odds ratio are considered, while other outcomes are seen as useful secondary information. Studies that only reported other outcomes but no CS rates and the CS odds ratio are not included.

### Study selection and data extraction

This study began with the selection of relevant publications’ titles and abstracts based on the searches' keywords. Studies that met the inclusion criteria were identified. In the case of duplicate studies, the most relevant or most recent publication was included.

Data extraction was performed independently by the researchers using a self-designed data collection form containing the following information for each study: publication date, design, participant/data type, intervention, sample size, measures, results, statistics, and effect on CS rate (significant decrease, significant increase, no significant effect, and limited its increase). Primary authors were contacted for clarification when there was missing information on study design or intervention characteristics.

### Methodological quality

Risk Of Bias In Non-randomized Studies - of Interventions (ROBINS-I) is a new tool for assessing the risk of bias in non-randomized studies for many kinds of organizational and public health interventions [27]. There are no randomized studies included in this systematic review. Thus, we used ROBINS-I to assess the risk of bias of each study. ROBINS-I was employed to assess the following aspects: a) confounders, b) selection of participants, c) classification of intervention, d) departure from intended interventions, e) missing data, f) measurement of outcomes, and g) selective reporting. Each part has five outcomes, namely, low risk of bias, moderate risk of bias, serious risk of bias, critical risk of bias, and no information [27]. The outcome of each study was evaluated with Grades of Recommendation, Assessment, Development and Evaluation (GRADE) using the GRADEpro Guideline Development Tool software [28], which categorized the quality or certainty of evidence into four levels: high, moderate, low, or very low.

## Results

### Characteristics of the included studies

**Fig. 2:**
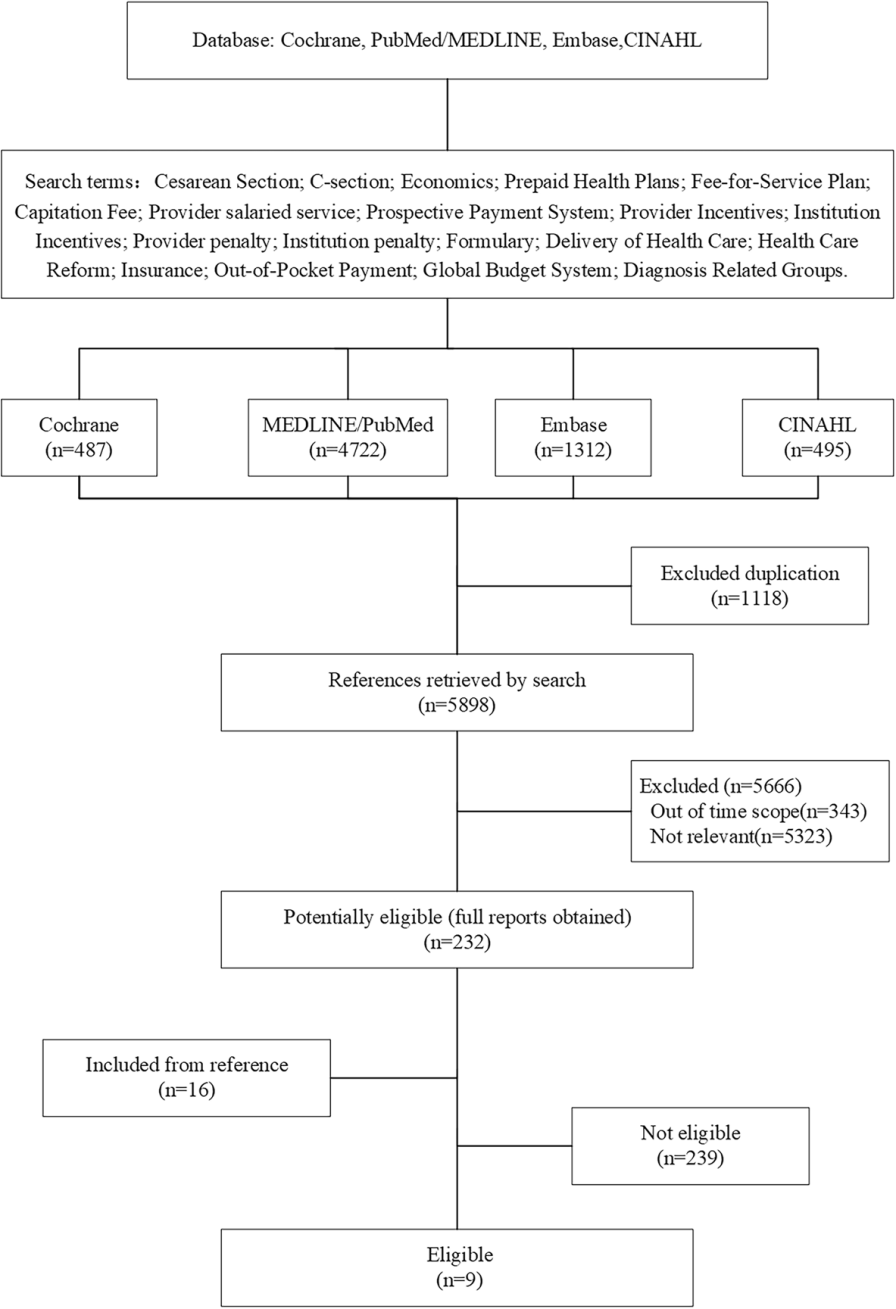
Flow Diagram of Study Selection

Our search found 5,898 articles: 5,666 were rejected after the initial screening, 343 were beyond the period of this study, and 5,323 did not report the CS rate or CS odds ratio. From the remaining 232 articles and 16 articles identified from reference lists, 239 were removed because their study designs were not about aiming to reduce the CS rate. Finally, nine studies that met all of this study’s criteria were included for the review (Fig. 2).

As shown in Table 1, there are no randomized controlled trials included in this study. The main design of included studies is ITS, and most studies were published in or after 2008. For the results of ROBINS-I, five studies had moderate risk of bias, three had serious risk of bias, and one had critical risk of bias. The results from GRADE are as follows: five studies were categorized as high certainty evidence, three studies as moderate certainty evidence, and another as low certainty evidence (Table 2). Seven focused on provider interventions, while two involved both provider and patient-side interventions (Table 3).

**Table 1:** Details of risk of bias of included studies using ROBINS-I

Author, year	Study design	Confounders	Selection of participant	Classification of intervention	Departure from intended interventions	Missing data	Measurement of outcomes	Selective reporting	Overall risk
Keeler and Fok, 1996 [29]	Interrupted time series	Moderate risk of bias	Low risk of bias	Serious risk of bias	No information	Low risk of bias	Low risk of bias	Low risk of bias	Serious risk of bias
Lo, 2008 [30]	Interrupted time series	Moderate risk of bias	Low risk of bias	Low risk of bias	No information	Low risk of bias	Low risk of bias	Low risk of bias	Moderate risk of bias
Misra, 2008 [31]	Cohort (one group pre + post (before and after)	Moderate risk of bias	Low risk of bias	Low risk of bias	No information	Low risk of bias	Low risk of bias	Low risk of bias	Moderate risk of bias
Hong and Linn, 2012 [32]	Interrupted time series	Moderate risk of bias	Serious risk of bias	Low risk of bias	No information	Low risk of bias	Low risk of bias	Low risk of bias	Serious risk of bias
Liu et al., 2013 [33]	Interrupted time series	Critical risk of bias	Serious risk of bias	Low risk of bias	No information	Low risk of bias	Low risk of bias	Low risk of bias	Critical risk of bias
Chen et al., 2014 [34]	Interrupted time series	Moderate risk of bias	Low risk of bias	Low risk of bias	No information	Low risk of bias	Low risk of bias	Low risk of bias	Moderate risk of bias
Kim et al., 2016 [35]	Cohort (one group pre + post (before and after)	Moderate risk of bias	Low risk of bias	Low risk of bias	No information	Low risk of bias	Low risk of bias	Low risk of bias	Moderate risk of bias
Liu et al., 2018 [36]	Cohort (one group pre + post (before and after)	Moderate risk of bias	Low risk of bias	Serious risk of bias	No information	Low risk of bias	Low risk of bias	Low risk of bias	Serious risk of bias
Kozhimannil, 2018 [37]	Cohort analytic (two group pre + post)	Moderate risk of bias	Low risk of bias	Low risk of bias	No information	Low risk of bias	Low risk of bias	Low risk of bias	Moderate risk of bias

**Table 2:** GRADE evidence of included studies

Author, year	Certainty assessment						Certainty (GARDE)
	Study design	Risk of bias	Inconsistency	Indirectness	Imprecision	Other considerations	
Keeler and Fok, 1996 [29]	Interrupted time series	serious	single study	not serious	not serious	none	⨁⨁⨁⊖
							MODERATE
Lo, 2008 [30]	Interrupted time series	not serious	single study	not serious	not serious	none	⨁⨁⨁⨁
							HIGH
Misra, 2008 [31]	Cohort (one group pre + post (before and after)	not serious	single study	not serious	not serious	none	⨁⨁⨁⨁
							HIGH
Hong and Linn, 2012 [32]	Interrupted time series	serious	single study	not serious	not serious	none	⨁⨁⨁⊖
							MODERATE
Liu et al., 2013 [33]	Interrupted time series	critical serious	single study	not serious	not serious	none	⨁⨁ ⊖ ⊖
							LOW
Chen et al., 2014 [34]	Interrupted time series	not serious	single study	not serious	not serious	none	⨁⨁⨁⨁
							HIGH
Kim et al., 2016 [35]	Cohort (one group pre + post (before and after)	serious	single study	not serious	not serious	none	⨁⨁⨁⨁
							HIGH
Liu et al., 2018 [36]	Cohort (one group pre + post (before and after)	serious	single study	not serious	not serious	none	⨁⨁⨁⊖
							MODERATE
Kozhimannil, 2018 [37]	Cohort analytic (two group pre + post)	not serious	single study	not serious	not serious	none	⨁⨁⨁⨁
							HIGH

**Table 3:** Summary of financial interventions for reducing the caesarean section rate

Author, Year	Intervention (effect)	Details of Intervention Strategies	Sample Size	Outcome Measure	Certainty (GARDE)
Keeler and Fok, 1996 [29]	Provider intervention: fee equalization for hospitals (No significant effect)	Fees for vaginal deliveries were increased by 3%, and fees for CS were reduced to the amount charged for vaginal deliveries (an average reduction of 18%).	11,767	CS rate (%) Before intervention 25.3 Post intervention 24.6 *P* > 0.05	⨁⨁⨁⊖ MODERATE
Lo, 2008 [30]	Provider intervention: fee equalization for hospitals (No significant effect)	Fees for vaginal birth after caesarean section were raised to the level of caesarean section in April 2003; Fees for vaginal deliveries were also raised to the level of caesarean section in May 2005.	1,084,686	OR (95%CI) for CS Post-VBAC fee rose 1.05 (1.00–1.09) Post-fee equivalence 1.03 (0.97–1.09)	⨁⨁⨁⨁ HIGH
Hong and Linn, 2012 [32]	Provider intervention: fee equalization for hospitals (No significant effect)	The payment to hospitals for vaginal deliveries was raised in May 2005. Before this intervention, the payment for caesarean section with medical indications was $911 to $1132 but the payment for vaginal deliveries was only $506 to $609. After this intervention, the payment for deliveries was $911 to $1132 regardless of delivery mode.	51,085	OR (95%CI) for CS ^a^Unplanned CS 0.978 (0.90–1.07) ^a^Planned CS 0.995 (0.09–1.06) CDMR 0.862 (0.69–1.07)	⨁⨁⨁⊖ MODERATE
	Patient intervention: co-payment for CDMR for patients (No significant effect)	Between May 2005 and May 2006, the payment for CDMR was equal to that for vaginal deliveries ($911 to $1132). After the co-payment policy applied, the payment for CDMR was $506 and the co-payment for that was increased from $0 to $475–697.		OR (95%CI) for CS ^a^Unplanned CS 0.960 (0.88–1.05) ^a^Planned CS 0.942 (0.88–1.00) CDMR 1.083 (0.87–1.35)	
Liu et al., 2013 [33]	Provider intervention: ^b^the global budget system for hospitals (No significant effect)	Fees for services plan were replaced by the global budgeting system at this tertiary hospital in July 2002 for controlling CS rates. The global budget system is prospective payment system, which aiming at allocating resource and controlling cost.	35,616	CS rate (%) Before GBS 35.1 After GBS 36.7 *P* = 0.0525	⨁⨁ ⊖ ⊖ LOW
Chen et al., 2014 [34]	Provider intervention: fee equalization for hospitals (No significant effect)	A global fee ($905–1132) was set for obstetric services at different levels of medical institutions, regardless of the mode of delivery in May 2005.	1,003,412	OR for CS 20–1.033 25–1.009 30–0.987 35–0.966 40–0.944 45–0.923	⨁⨁⨁⨁ HIGH
	Patient intervention: co-payment for CDMR for patients (No significant effect)	After May 2006, the Bureau of National Health Insurance (BNHI) took partial reimbursement for where mothers had to pay a co-payment. With this intervention, physicians still received the same total payments for CDMR, but their payments came from two components. For instance, in medical centers, physicians obtain payments of $1132 for CDMR, which included the reimbursement of $609 for vaginal delivery and the copayment of $523, paid by the BNHI and mothers, respectively.		OR for CS 20–1.037 25–1.021 30–1.007 35–0.992 40–0.977 45–0.961	
Liu et al., 2018 [36]	Provider intervention: ^c^case payment for hospitals (Significant increase)	Insurance agencies took a payment reform in 2009 and completed in 2011, the traditional fee-for-service payment was transformed into case payment for caesarean sections. The case compensation standard for CS is higher than that of vaginal deliveries ($493.47 for CS without complications, $197.39 for vaginal deliveries without complications).	28,314	CS rate (%) Pre-CPR:26.124% Post-CPR:32.475% *P* < 0.001	⨁⨁⨁⊖ MODERATE
Misra, 2008 [31]	Provider intervention: ^d^risk-adjusted capitation for hospitals (Limited its increase)	In 1997, the Maryland Department of Health and Mental Hygiene replaced the mixed model of fee-for-service and voluntary managed care enrollment for a majority of Medicaid enrollees with a mandatory managed care system called HealthChoice. HealthChoice capitation rates are risk-adjusted. The monthly payments to a managed care organization for providing all necessary services to a particular enrollee are based on the individual’s documented health status. Managed care organizations receive a higher payment when the more severe the patients’ clinical conditions.	128,743	OR (95%CI) for CS Primary CS 0.67 (0.573~0.774) Repeat CS 0.71 (0.623~0.804)	⨁⨁⨁⨁ HIGH
Kim et al., 2016 [35]	Provider intervention: ^e^ the diagnosis-related group payment for hospitals (Significant decrease)	Vaginal deliveries followed the fee-for-service system and the cost for CS followed the diagnosis-related group payment system. In 2002, the diagnosis-related group payment system for caesarean section only in voluntary health sectors. From July 2012, he diagnosis-related group payment system became mandatory in hospital and clinics, the it was also applied to general hospitals and tertiary hospitals since July 2013.	1,289,989	OR (95%CI) for CS Mandatory adoption of DRG system 0.823 (0.816–0.830) A longer length of the DRG system adoption period 0.997 (0.996–0.998)	⨁⨁⨁⨁ HIGH
Kozhimannil, 2018 [37]	Provider intervention: fee equalization for hospitals and clinicians (Significant decrease)	Before this intervention, facility fees were $3144 and $5266 for uncomplicated vaginal and cesarean births, respectively. After intervention, the policy changed the rate to $3528 for uncomplicated births regardless of the mode of delivery. Professional services fees also changed because of the policy, from $776.62 and $1147.42 for prenatal, delivery, and postpartum care for uncomplicated vaginal and cesarean births, respectively, to a single blended rate of $867.37.	671,177	CS rate (%) Intervention-group: decrease 3.24% Control-group: increase 0.6%	⨁⨁⨁⨁ HIGH

Note:

^a^ Unplanned caesarean sections and planned caesarean sections are types of caesarean section with medical indications

^b^ Global Budget System is one of prospective reimbursement for healthcare providers and government set a target on the amount of overall cost for health providers

^c^ Case payment means that healthcare provider will get a fixed price per admission irrespective of the actual health cost incurred

^d^ Risk-adjustment capitation was charged monthly according to the applicants’ health status, and managed care organizations receive a higher payment, the more severe the patients’ clinical conditions

^e^ Diagnostic-related group payments means groups of patients with similar clinical conditions and these patients would incur comparable health costs

### Provider-side interventions

Based on the assumption that a higher fee for CS could lead to an increase in CS abuse by healthcare providers, three studies [29, 30, 37] reported on attempts at controlling CS rates through equalizing fees, including facility fees and professional fees. Facility fees are often charged in healthcare settings to cover operation costs [38], while professional fees are charged by doctors for medical services they provide to patients [39]. Keeler [29] and Lo [30] showed that fee-equalizing for facilities had no significant effect on reducing CS rates. In the first case, California Blue Cross increased the fee by 3% for vaginal deliveries and reduced the fee by 18% for CS to decrease the CS rate in 1993 (moderate certainty evidence) [29]. In the second case, aiming to reduce the CS rate, the National Health Insurance of Taiwan raised the fee for vaginal birth after a CS (VBAC) to the level of CS since April 2003, following which all fees for vaginal deliveries were raised to the level of CS in May 2005 (high certainty evidence) [30]. It is worth noting that another high certainty evidence from Kozhimannil [37] showed that an equality fee intervention conducted by Minnesota’s Medicaid Program, which raised both facility fees and professional fees, significantly decreased the CS rate.

Four of the studies examined the effect of payment reform for reducing the CS rate [31, 33, 35, 36]. In Taiwan, China, Liu et al. [33] evaluated the effectiveness of the hospital global budget system (GBS) reform to reduce the caesarean section rates in a tertiary hospital was uncertain in 2002 (low certainty evidence). In Henan, China, Liu et al. [36] described that payment reform from 2009 to 2011 that transforming a fee-for-service payment policy into a case payment policy has the opposite effect of increased the CS rate (moderate certainty evidence). However, two high-certainty studies revealed that risk-adjusted payment could be effective for controlling the CS rate [31, 35]. First, Misra [31] described the effect of provider intervention with risk-adjusted capitation for CS in Maryland, USA. In this high certainty of evidence study, capitation was charged monthly according to the applicants’ health status, which could limit increases of the CS rate [31]. Second, Kim et al. [35] showed that a diagnosis-related group payment system for CS versus fee-for-service system for vaginal deliveries was effective in reducing the CS rate in Korea.

### Both provider and patient-side interventions

Two studies [32, 34] examined a financial intervention strategy in which provider intervention was combined with patient intervention. In both studies, the provider-side intervention involved equalizing fees, for example, the physicians in medical centers would receive a payment of $911 to $1,132 regardless of delivery mode (vaginal delivery, caesarean section with medical indications, or CDMR) [32, 34]. The intervention on the patient-side was a co-payment policy for elective CS. For example, physicians would receive a payment of $1,203 for a CDMR in medical centers once the co-payment policy was implemented, which included a reimbursement of $506 from the Nation Health Insurance and a co-payment of $697 from the patients, respectively [32, 34]. However, these two studies (one was of high certainty and the other of moderate certainty) showed both provider and patient-side incentives had no significant effect in reducing the CS rate.

## Discussion

This systematic review examined the effectiveness of patient-side and provider-side financial intervention strategies in limiting unnecessary CS.

With regard to the patient-side intervention, two existing studies revealed that co-payment had no significant effect on reducing the CS rate. This was consistent with previous studies [40]. However, expectant mothers may influence the delivery mode. For instance, some may fear pain during labor, have a belief in the deteriorating quality of care during labor and vaginal birth [40, 41], and be willing to pay for CS [32]. However, doctors play a more important role than mothers do in the selection of delivery mode [42, 43], and mothers’ preference of delivery mode is unlikely to be a major driver of high CS rates [40]. Thus, we do not recommend a co-payment policy to reduce CS rates without strong evidence supporting its effectiveness.

With regard to provider-side interventions, previous studies show that financial interventions could influence the behavior of physicians by promoting antibiotic prescribing practices [44], improving the provision of necessary healthcare [45], and improving clinical care quality [46]. Theoretically, financial incentive strategies could be effective in reducing the CS rate, especially the rate of CDMR. However, this intervention was not as effective as was expected.

Financial incentives for provider-side intervention in this systematic review comprise equalizing fees, case payment, national healthcare policy of the GBS, diagnosis-related group payment system for CS, and risk-adjusted capitation for CS.

Simply equalizing fees for facilities was not effective in the existing literature for a few possible reasons: (1) CS is regarded as defensive medicine for avoiding medical lawsuits [47]; (2) the medical professionals’ demand for leisure was positively associated with CS utilization because performing a CS is faster than a vaginal delivery, resulting in more leisure time for medical care providers [48, 49]; and (3) lack of guidance from behavioral psychology and behavioral economics resulted in the creation of an ineffective financial intervention strategy [50]. In short, a simple economic incentive intervention appears to be less effective in influencing physicians’ delivery mode decision than expected. Evidence from Taiwan, China suggested that the method of equalizing fees did not work at all. Medical resource consumption of a CS is generally higher than that of a vaginal delivery. However, even once the price of a vaginal delivery was raised to the level of a CS [32, 34], it still had no significant effect in reducing the CS rate. Therefore, equalizing fees for facilities alone did not appear to reduce the CS rate.

Evidence from Henan, China showed that case payment was not effective for reducing the CS rate, which could relate to unreasonable case compensation standards for CS ($493.47), which is much higher than vaginal delivery ($197.39) [36]. Thus, the healthcare setting and physicians may prefer CS over vaginal deliveries for financial reasons [51].

However, it appears that the risk-adjusted payment methods such as a diagnosis-related group payment system for CS and a risk-adjusted capitation for CS were effective in controlling the CS rate [31, 33]. The potential reason is that a risk-adjusted payment system introduces competition among healthcare service providers [52]. The risk-adjusted price is based on competitive forces with other hospitals [53], with the healthcare setting receiving a risk-adjusted payment. Moreover, hospitals and clinics will incur a significant loss if physicians perform unnecessary medical care services such as CDMR [54, 55]. In addition, since a risk-adjusted payment system functions to improve cost management of the hospital [56], it will be advantageous to train and educate physicians to provide only medically necessary services.

Additionally, the unprecedented rapid increase of CS utilization rates is multifactorial. It includes behavioral, psychosocial, organizational, and financial factors of women, families, healthcare professionals, and healthcare organizations and systems [20]. Many related stakeholders influence the effectiveness of interventions. Working with different intervention priorities and interests poses a barrier for effective intervention implementation. Interventions that are single-component or that address the concerns/needs of one of the stakeholders without considering the others are not ideal and are more likely to fail. Therefore, policymakers must consider the interests of all stakeholders. In other words, based on the perspective of multi-interest groups, policymakers can find a “sensitive and cost-effective point” to reduce irrational CS utilization, and develop and implement corresponding strategies to guarantee the effectiveness of the financial intervention. The high certainty evidence from Minnesota’s Medicaid Program is a good example of how an intervention policy that takes into account the personal interests of doctors by equalizing fees for both facilities and physicians could significantly decrease the CS rate [37].

## Limitations and strengths of the review

We believe this is the first global study to focus on the effectiveness of various financial intervention strategies in reducing unnecessary CS. There are several limitations to the interpretation of our findings. First, existing studies are sparse and limited. Because there are too few studies in each sub-intervention group, as well as the diversity study design among sub-groups, we were unable to undertake the sub-analyses as we had planned. Second, lack of direct comparative studies made it difficult for us to point out which could be the best financial interventions for reducing the CS rate. Third, although all the studies were about reducing unnecessary CS, the clinical data about the appropriateness of the CS conducted were unavailable. Therefore, there is no basis for us to distinguish and assess whether the reduced number of CS were all unnecessary ones. Fourth, the impact of financial interventions on reducing CS is quite complex. Thus, differences in the studies may have resulted from unaccounted for differences in countries, policy environments, target population groups, and variables measured.

Future research using randomized controlled designs or fixed effect modeling longitudinal studies can provide more robust predictions regarding the effect of financial interventions on reducing unnecessary CS. Finally, the systematic review methodology and use of the ROBINS-I tool are the strengths of the review.

## Conclusions

Although there is still a paucity of high-quality research on this topic, based on current evidence, we can draw three conclusions. First, although we can’t draw the conclusion that the risk-adjusted payment methods such as a diagnosis-related group payment system for CS and a risk-adjusted capitation for CS are effective provider-side interventions. However, the effectiveness of these risk-adjusted payment methods looks promising; thus, strong evidence is needed for proving that the provider-side intervention could be considered and is effective. Second, financial interventions should take stakeholders’ characteristics into account, especially doctors’ personal interests. Third, high quality RCT data and direct comparative studies on different financial interventions in the future could confirm or refute the outcomes of the existing research.

## Additional file

Additional file 1: Full electronic search strategy used in the systematic review; We conducted a systematic search of English language CS rate relevant articles following this search strategy. (DOCX 21 kb)

## Data Availability

The authors confirm that all data is contained within the manuscript and its additional files.

## Abbreviations

CDMR: Caesarean delivery on maternal request
CS: Cesarean section
GRADE: Grades of Recommendation, Assessment, Development, and Evaluation
HCE: Certainty evidence
ITS: Interrupted time series
LCE: Low certainty evidence
MCE: Moderate certainty evidence
RCT: Randomized control trial
ROBINS-I: Risk of Bias in Non-randomized Studies - of Interventions
VBAC: Vaginal birth after a cesarean section
WHO: World Health Organization
