# Supplementary material for: The effectiveness of financial intervention strategies for reducing caesarean section rates: a systematic review
Source: BMC Public Health. 2019 Aug 9;19:1080. doi: 10.1186/s12889-019-7265-4 (PMC6688325; doi:10.1186/s12889-019-7265-4)
Supplement: Supplementary file 1 — Full electronic search strategy used in the systematic review; We conducted a systematic search of English language CS rate relevant articles following this search strategy. (DOCX 21 kb) [file 12889_2019_7265_MOESM1_ESM.docx]

***Additional file 1***

***File name: Full electronic search strategy.docx***

***Title of data: Full electronic search strategy used in the systematic review***

***Database ONE: PubMed/Medline***

| #1 | (Cesarean Section[MeSH Terms]) OR Cesarean Section, Repeat[MeSH Terms] |
| --- | --- |
| #2 | ((((Cesarean Section[Title/Abstract]) OR C-section[Title/Abstract]) OR caesarean birth[Title/Abstract]) OR cesarean delivery[Title/Abstract]) OR caesarean[Title/Abstract] |
| #3 | ((Economics[MeSH Terms]) OR Economics[Title/Abstract]) OR economic[Title/Abstract] |
| #4 | (Reimbursement Mechanisms[MeSH Terms]) OR Reimbursement Mechanisms[Title/Abstract] |
| #5 | (Prepaid Health Plans[MeSH Terms]) OR Prepaid[Title/Abstract] |
| #6 | (((Fee-for-Service Plan[MeSH Terms]) OR Fee-for-Service Plan[Title/Abstract]) OR Fee for Service[Title/Abstract]) OR Fees for Service[Title/Abstract] |
| #7 | (Capitation Fee[MeSH Terms]) OR Capitation[Title/Abstract] |
| #8 | Provider salaried service[Title/Abstract] |
| #9 | (Prospective Payment System[MeSH Terms]) OR Prospective Payment System[Title/Abstract] |
| #10 | Provider incentives[Title/Abstract] |
| #11 | Institution incentives[Title/Abstract] |
| #12 | (Provider grant[Title/Abstract]) AND Provider allowance[Title/Abstract] |
| #13 | (Institution grant[Title/Abstract]) AND Institution allowance[Title/Abstract] |
| #14 | Provider penalty[Title/Abstract] |
| #15 | Institution penalty[Title/Abstract] |
| #16 | Formulary[Title/Abstract] |
| #17 | Patient incentives[Title/Abstract] |
| #18 | (Patient grant[Title/Abstract]) OR Patient allowance[Title/Abstract] |
| #19 | Patient penalty[Title/Abstract] |
| #20 | ((Delivery of Health Care[MeSH Terms]) OR Delivery of Health Care, Integrated[MeSH Terms]) OR Health Care delivery[Title/Abstract] |
| #21 | ((Health Care Reform[MeSH Terms]) OR Health Care Reform[Title/Abstract]) OR Medical Care Reform[Title/Abstract] |
| #22 | (Insurance[MeSH Terms]) OR Insurance[Title/Abstract] |
| #23 | ((Out-of-Pocket Payment[MeSH Terms]) OR Out-of-Pocket Payment[Title/Abstract]) OR OOP payment[Title/Abstract] |
| #24 | (global budget payment[Title/Abstract]) OR global budget system[Title/Abstract] |
| #25 | Search (((((((((((((Diagnosis Related Groups[MeSH Terms]) OR Diagnosis-Related Group[MeSH Terms]) OR Group, Diagnosis-Related[MeSH Terms]) OR Groups, Diagnosis-Related[MeSH Terms]) OR Groups, Diagnostic-Related[MeSH Terms]) OR Groups, Diagnostic Related[MeSH Terms]) OR Diagnostic-Related Group[MeSH Terms]) OR Diagnostic Related Group[MeSH Terms]) OR Diagnostic-Related Groups[MeSH Terms]) OR Group, Diagnostic-Related[MeSH Terms]) OR Group, Diagnostic Related[MeSH Terms]) OR DRGs[MeSH Terms]) OR Case Mix[MeSH Terms]) OR Case Mixes[MeSH Terms] |
| #26 | #1 OR #2 |
| #27 | #3 OR #4 OR #5 OR #6 OR #7 OR #8 OR #9 OR #10 OR #11 OR #12 OR #13 OR #14 OR #15 OR #16 OR #17 OR #18 OR #19 OR #20 OR #21 OR #22 OR #23 OR #24 OR #25 |
| #28 | #26 AND #27 |

***Database Two: Embase***

| #1 | 'cesarean section':ab,ti OR 'c-section':ab,ti OR 'caesarean birth':ab,ti OR 'cesarean delivery':ab,ti OR 'caesarean':ab,ti |
| --- | --- |
| #2 | 'economics':ab,ti OR 'economic':ab,ti |
| #3 | reimbursement mechanism':ab,ti |
| #4 | fee-for-service':ab,ti OR 'fees for service':ab,ti |
| #5 | prepaid health plans':ab,ti OR 'prepaid':ab,ti |
| #6 | capitation fee':ab,ti OR 'capitation':ab,ti |
| #7 | provider salaried service':ab,ti |
| #8 | prospective payment system':ab,ti |
| #9 | provider incentives':ab,ti |
| #10 | institution incentives':ab,ti |
| #11 | provider grant':ab,ti OR 'provider allowance':ab,ti |
| #12 | institution grant':ab,ti OR 'institution allowance':ab,ti |
| #13 | provider penalty':ab,ti |
| #14 | institution penalty':ab,ti |
| #15 | formulary':ab,ti |
| #16 | premium':ab,ti |
| #17 | co-payment':ab,ti |
| #18 | patient incentives':ab,ti |
| #19 | patient grant':ab,ti OR 'patient allowance':ab,ti |
| #20 | patient penalty':ab,ti |
| #21 | health care delivery':ab,ti OR 'delivery of health care':ab,ti |
| #22 | health care reform':ab,ti OR 'medical care reform':ab,ti |
| #23 | insurance':ab,ti |
| #24 | out-of-pocket payment':ab,ti OR 'oop payment':ab,ti |
| #25 | global budget payment':ab,ti OR 'global budget system':ab,ti |
| #26 | diagnosis related groups':ab,ti OR 'diagnosis-related group':ab,ti OR 'drg':ab,ti OR 'drgs':ab,ti |
| #27 | #2 OR #3 OR #4 OR #5 OR #6 OR #7 OR #8 OR #9 OR #10 OR #11 OR #12 OR #13 OR #14 OR #15 OR #16 OR #17 OR #18 OR #19 OR #20 OR #21 OR #22 OR #23 OR #24 OR #25 OR #26 |
| #28 | #1 AND #27 |

***Database Three: CINAHL***

| #1 | TI Cesarean Section OR TI C-section OR TI caesarean birth OR TI cesarean delivery OR TI caesarean OR AB Cesarean Section OR AB C-section OR AB caesarean birth OR AB cesarean delivery OR AB caesarean |
| --- | --- |
| #2 | AB Economics OR TI Economics OR AB economic OR TI economic |
| #3 | TI ( "Fee for Service" OR "Fees for Service" ) OR AB ( "Fee for Service" OR "Fees for Service" ) |
| #4 | AB Prepaid OR TI Prepaid |
| #5 | TI Provider salaried service OR AB Provider salaried service |
| #6 | TI Prospective payment OR AB Prospective payment |
| #7 | TI Provider incentives OR AB Provider incentives OR TI Institution incentives OR AB Institution incentives |
| #8 | TI Provider grant OR AB Provider grant OR TI Provider allowance OR AB Provider allowance |
| #9 | TI Institution grant OR AB Institution grant OR TI Institution allowance OR AB Institution allowance |
| #10 | TI Provider penalty OR AB Provider penalty OR TI Institution penalty OR AB Institution penalty |
| #11 | TI Formulary AND AB Formulary |
| #12 | TI Premium OR AB Premium |
| #13 | TI Co-payment OR AB Co-payment |
| #14 | TI Patient incentives OR AB Patient incentives |
| #15 | TI Patient grant OR AB Patient grant OR TI Patient allowance OR AB Patient allowance |
| #16 | TI Health Care delivery OR AB Health Care delivery OR TI delivery of Health Care OR AB delivery of Health Care |
| #17 | TI insurance OR AB insurance |
| #18 | TI OOP payment OR AB OOP payment OR TI out-of-pocket payment OR AB out-of-pocket payment |
| #19 | TI global budget payment OR AB global budget payment OR TI global budget system OR AB global budget system |
| #20 | TI diagnosis related groups OR AB diagnosis related groups OR TI diagnosis-related group OR AB diagnosis-related group OR TI drgs OR AB drgs OR TI drg OR AB drg |
| #21 | #2 OR #3 OR #4 OR #5 OR #6 OR #7 OR #8 OR #9 OR #10 OR #11 OR #12 OR #13 OR #14 OR #15 OR #16 OR #17 OR #18 OR #19 OR #20 |
| #22 | #1 AND #21 |

***Database Three: Cochrane Library***

| #1 | (Cesarean Section):ti,ab,kw OR (C-section):ti,ab,kw OR (caesarean birth):ti,ab,kw OR (cesarean delivery):ti,ab,kw OR (caesarean):ti,ab,kw |
| --- | --- |
| #2 | (Economics):ti,ab,kw OR (economic):ti,ab,kw |
| #3 | (reimbursement mechanisms):ti,ab,kw OR (reimbursement mechanism):ti,ab,kw |
| #4 | (Prepaid Health Plans):ti,ab,kw OR (Prepaid):ti,ab,kw |
| #5 | (Fee for Service):ti,ab,kw OR (Fees for Service):ti,ab,kw |
| #6 | (Capitation Fee):ti,ab,kw OR (Capitation):ti,ab,kw |
| #7 | (Provider salaried service):ti,ab,kw |
| #8 | (Prospective payment):ti,ab,kw |
| #9 | (Provider incentives):ti,ab,kw OR (Institution incentives):ti,ab,kw |
| #10 | (Provider grant):ti,ab,kw OR (Provider allowance):ti,ab,kw |
| #11 | (Institution grant):ti,ab,kw OR (Institution allowance):ti,ab,kw |
| #12 | (Provider penalty):ti,ab,kw |
| #13 | (Institution penalty):ti,ab,kw |
| #14 | (Formulary):ti,ab,kw |
| #15 | (Patient incentives):ti,ab,kw |
| #16 | (Patient grant):ti,ab,kw OR (Patient allowance):ti,ab,kw |
| #17 | (Patient penalty):ti,ab,kw |
| #18 | (Delivery of Health Care):ti,ab,kw OR (Delivery of Health Care):ti,ab,kw OR (Health Care delivery):ti,ab,kw |
| #19 | (Health Care Reform):ti,ab,kw OR (Medical Care Reform):ti,ab,kw |
| #20 | (Insurance):ti,ab,kw |
| #21 | (Out-of-Pocket Payment):ti,ab,kw OR (OOP payment):ti,ab,kw |
| #22 | (global budget payment):ti,ab,kw |
| #23 | (Diagnosis Related Groups):ti,ab,kw OR (Diagnosis-Related Group):ti,ab,kw OR (DRG):ti,ab,kw OR (DRGs):ti,ab,kw |
| #24 | #2 OR #3 OR #4 OR #5 OR #6 OR #7 OR #8 OR #9 OR #10 OR #11 OR #12 OR #13 OR #14 OR #15 OR #16 OR #17 OR #18 OR #19 OR #20 OR #21 OR #22 OR #23 |
| #25 | #1 AND #24 |
